# A comprehensive Hirshfeld and iterative-Hirshfeld dataset for 5080 inorganic materials from first-principles calculations

**DOI:** 10.1016/j.dib.2026.113228

**Published:** 2026-09-04

**Authors:** Tianhao Su, Musen Li, Xitao Wang, Shunbo Hu, Tong-Yi Zhang

**Affiliations:** Material Genome Institute, Shanghai University, Shanghai, 200444, China

**Keywords:** Hirshfeld charge, Iterative Hirshfeld, Inorganic materials, Density functional theory

## Abstract

Atomic charge descriptors are widely used to interpret chemical bonding, quantify charge transfer, and construct data-driven models for inorganic materials. This data article describes a first-principles dataset containing Hirshfeld and iterative-Hirshfeld outputs for 5080 inorganic materials, corresponding to 31,288 atomic sites and 77 chemical elements. For each material, the database stores the atomic structure, Materials Project identifier, calculation metadata, standard Hirshfeld charges, iterative Hirshfeld charges, Hirshfeld relative volumes, and iterative-Hirshfeld relative volumes. The data were generated using density-functional theory calculations in VASP with a consistent local-density-approximation setup, a 500 eV plane-wave cutoff, 0.25 inverse angstrom k-point spacing, and an electronic convergence threshold of 1e-6 eV. The dataset is distributed as an ASE database with accompanying JSON statistics, analysis scripts, figures, and a Flask-based web interface. It can support charge-transfer analysis, benchmarking of charge-partitioning methods, and charge-informed machine-learning workflows in which atomic populations, charge-transfer magnitudes, or volume descriptors are used as features for materials-property prediction.

Specifications Table

**Table:** 

Subject	Engineering & Materials science
Specific subject area	Atomic charge analysis and volume partitioning in inorganic crystalline materials.
Type of data	Raw and processed data; ASE SQLite database; JSON metadata and statistics; CSV element-coverage table; PNG figures; Python scripts; Flask web-interface package.
Data collection	The data were generated from VASP self-consistent-field calculations using a consistent LDA setup and Hirshfeld analysis. Archived entries were included when they had a Materials Project identifier, completed SCF status, four complete Hirshfeld-related arrays, and array lengths matching the number of atomic sites.
Data source location	*Structures are indexed by Materials Project identifiers stored as mpid. Data were generated at Shanghai University and curated through the GitHub/Zenodo repository.*
Data accessibility	Repository name: https://github.com/iamusen/hirshchg.git Data identification number: *10.5281/zenodo.21166566* Direct URL to data: https://doi.org/10.5281/zenodo.21166566 Instructions for accessing these data: *download the archived GitHub release from Zenodo and read data/h.db with ASE, or use the packaged Flask web interface*.
Related research article	No.

## 1 Value of the Data

• The dataset provides site-resolved Hirshfeld and iterative-Hirshfeld quantities for 5080 inorganic materials in a reusable database format, reducing the need for repeated first-principles charge-partitioning calculations.
• The combination of standard Hirshfeld charges, iterative Hirshfeld charges, and the corresponding relative-volume descriptors enables users to compare charge-partitioning outputs across the same set of structures and to select descriptors appropriate for different modeling tasks.
• The 31,288 atomic-site records cover 77 elements and can be merged with Materials Project identifiers for studies that connect atomic charge descriptors with crystal chemistry, composition, stability, transport, or electronic properties.
• The data can be used to construct charge-informed materials-informatics features, such as element-resolved charge statistics, charge-transfer magnitudes, cation/anion charge distributions, or structure-level moments of atomic-charge populations, complementing composition- and structure-based descriptors used in machine-learning studies.
• The ASE database, validation scripts, JSON summaries, and Flask interface make the data usable both in automated screening workflows and by non-specialist users who need to search or inspect individual material entries without writing database queries.

## 2 Background

Hirshfeld population analysis partitions an electronic density into atom-associated contributions using reference atomic densities [1], and iterative Hirshfeld analysis extends this idea through self-consistent updating of the reference populations [2]. These descriptors are commonly used as compact, site-resolved quantities for describing charge transfer and atom-in-material environments. Descriptor quality and availability are central to machine-learning studies in materials science, where curated features have been used to predict glass-transition temperatures, machining responses, shape-memory transformation temperatures, and perovskite lattice constants [[3], [4], [5], [6]]. A direct machine-learning application of Hirshfeld charges was demonstrated by Ko et al. [7], who trained a fourth-generation high-dimensional neural-network potential using DFT Hirshfeld charges as reference charge populations; the resulting charges were used to calculate electrostatic energies and as non-local inputs to the short-range energy model. Related work has modeled partial atomic charges in metal-organic frameworks [8]. Hirshfeld-derived volume information has also entered downstream machine-learning workflows: earlier models predicted Hirshfeld volume ratios for many-body-dispersion calculations, and Moerman et al. developed MBD-ML to predict related atom-resolved polarizability and C6 ratios for molecules and materials [9]. Although these dispersion targets are not atomic charges, they demonstrate that atom-resolved volume and response descriptors derived from density partitioning can serve as machine-learning targets and inputs to subsequent materials calculations. The present dataset was compiled to make a consistent set of Hirshfeld and iterative-Hirshfeld outputs available for inorganic materials indexed by Materials Project identifiers [10]. The data package was organized to support reuse through a standard ASE database [11], accompanying metadata files, validation scripts, summary figures, and a lightweight web interface.

## 3 Data Description

This article describes a database of Hirshfeld-related atomic descriptors for 5080 inorganic materials. Each row in the main database corresponds to one material and contains the atomic structure, the Materials Project identifier, calculation status fields, and four arrays stored in the ASE row.data dictionary: hirshfeld, hirshfeld_i, hirshfeld_relvol, and hirshfeld_i_relvol. The first two arrays contain atomic charges in units of the elementary charge. The latter two arrays contain dimensionless relative-volume ratios from the standard and iterative Hirshfeld procedures. The length of each array equals the number of atomic sites in the corresponding structure.

The main file, h.db, contains 5080 completed self-consistent-field calculations. The database includes 31,288 atomic sites, with material sizes ranging from 1 to 11 atoms per database entry and a median of 6 atoms. The database covers 77 elements, as visualized on the periodic table in Fig. 7. Oxygen is the most frequent element in the dataset, with 4165 atomic-site occurrences.

Table 1 summarizes the main database statistics. All 5080 rows contain the four required arrays, and every array length matches its structure. Material-level charge sums are neutral within floating-point precision; the maximum absolute residual is approximately 1.8 × 10^−14^ e.

**Table 1:** Database statistics.

Property	Value
Number of materials	5080
Number of atomic sites	31,288
Average atoms per material	6.16
Median atoms per material	6
Minimum atoms per material	1
Maximum atoms per material	11
Unique elements	77
Most common element	O, 4165 occurrences
Rows with all Hirshfeld-related arrays	5080 (100%)
Calculation status	5080 completed SCF calculations
Main database size	17 MB
Web-interface package size	4.7 MB

Table 2 gives descriptive statistics for the four stored atomic-site arrays. Standard Hirshfeld charges range from −0.733 e to 0.978 e, with a standard deviation of 0.234 e. Iterative Hirshfeld charges span a wider numerical range, from −19.584 e to 4.686 e, with a standard deviation of 1.864 e. The correlation coefficient between the standard and iterative Hirshfeld charge arrays is 0.674 over all 31,288 atomic sites. This moderate correlation and the wider iterative-Hirshfeld range indicate that the two charge definitions should not be treated as interchangeable. An explicit outlier audit found 511 sites with |hirshfeld_i| > 5 e, including 40 sites with |hirshfeld_i| > 10 e; no non-finite values were present. The minimum value, −19.5837 e, occurs for a Si site in mp-1224,819 (Ga3Ni4Si) and is paired with an iterative relative volume of 29.9532. Such extremes demonstrate strong method and reference-population sensitivity and must not automatically be interpreted as formal oxidation states, directly observable atomic charges, or evidence of physically larger charge transfer. Standard Hirshfeld values are the more conservative, lower-variance descriptors. Iterative-Hirshfeld values can be retained for method comparison or sensitivity-aware modeling, but users should inspect, transform, or robustly scale extreme values and validate conclusions within the relevant chemical domain. The relative-volume arrays are dimensionless and should not be interpreted as charges.

**Table 2:** Atomic-site statistics for the stored Hirshfeld-related arrays.

Field	Count	Minimum	Maximum	Mean	Standard deviation	Median	Q25	Q75
hirshfeld (e)	31,288	−0.733	0.978	0.000	0.234	−0.008	−0.171	0.120
hirshfeld_i (e)	31,288	−19.584	4.686	0.000	1.864	−0.025	−0.990	1.199
hirshfeld_i_relvol	31,288	0.005	29.953	1.474	1.696	1.043	0.460	1.778
hirshfeld_relvol	31,288	0.389	1.272	0.990	0.058	0.999	0.962	1.029

Table 3 lists representative database entries. The rows illustrate the database schema rather than a filtered subset for a specific materials class. Each material entry stores a formula, an mpid, the number of atomic sites, and the material-level mean and standard deviation of the site-resolved Hirshfeld quantities.

**Table 3:** Representative material entries.

MPID	Formula	Atoms	Hirshfeld mean ± std (e)	Hirshfeld-I mean ± std (e)
mp-973,433	LuRu2Sc	4	0.0000 ± 0.1499	0.0000 ± 0.7605
mp-2350	LaS	2	0.0000 ± 0.1773	0.0000 ± 0.7348
mp-1080,455	Cu2Ti2As2Si2	8	0.0000 ± 0.1711	0.0000 ± 2.0556
mp-552,738	Bi2TmIO4	8	−0.0000 ± 0.3941	−0.0000 ± 2.0198
mp-517	Ta3Si6	9	−0.0000 ± 0.1188	0.0000 ± 1.2244
mp-12,776	FeTlS2	4	−0.0000 ± 0.1775	−0.0000 ± 0.7616
mp-1009,009	LiF	2	0.0000 ± 0.1819	0.0000 ± 0.9898
mp-7	S6	6	−0.0000 ± 0.0000	0.0000 ± 0.0000
mp-1077,033	Yb2Se4	6	−0.0000 ± 0.1151	0.0000 ± 2.1808
mp-1039,305	BiMg	2	0.0000 ± 0.1334	0.0000 ± 1.3629

Table 4 reports the 20 most frequent elements in the atomic-site population. These values are useful for users selecting subsets with sufficient coverage for element-specific analysis or model training. The complete element-coverage table is provided as data/element_coverage.csv; it lists all 77 covered elements, their atomic-site counts, percentages relative to all 31,288 sites, material-level occurrence counts, and representative material examples Table 5.

**Table 4:** Top 20 elements by atomic-site occurrence.

Rank	Element	Occurrences	Percentage of all sites (%)
1	O	4165	13.3
2	Mg	1151	3.7
3	F	1126	3.6
4	S	1094	3.5
5	N	849	2.7
6	Li	798	2.6
7	Si	774	2.5
8	Se	773	2.5
9	Ge	764	2.4
10	Cu	707	2.3
11	Te	685	2.2
12	Ni	586	1.9
13	Ga	550	1.8
14	C	540	1.7
15	Pd	533	1.7
16	Na	527	1.7
17	Ag	508	1.6
18	Mn	504	1.6
19	La	488	1.6
20	Sn	480	1.5

**Table 5:** VASP settings used for data generation.

Parameter	Value
ENCUT	500.0 eV
KSPACING	0.25 inverse angstrom
EDIFF	1 × 10^−6^ eV
ALGO	All
PREC	Accurate
ISMEAR	0
SIGMA	0.02 eV
NELM	200
LASPH	True
IVDW	22
LCHARG	True
LWAVE	True
LH5	True
Pseudopotential set	PAW_PBE
Special setups	V: _sv; Ca: _sv; Ti: _sv
VASP version	6.6.0 (build 7 April 2026)

**Fig. 1:**
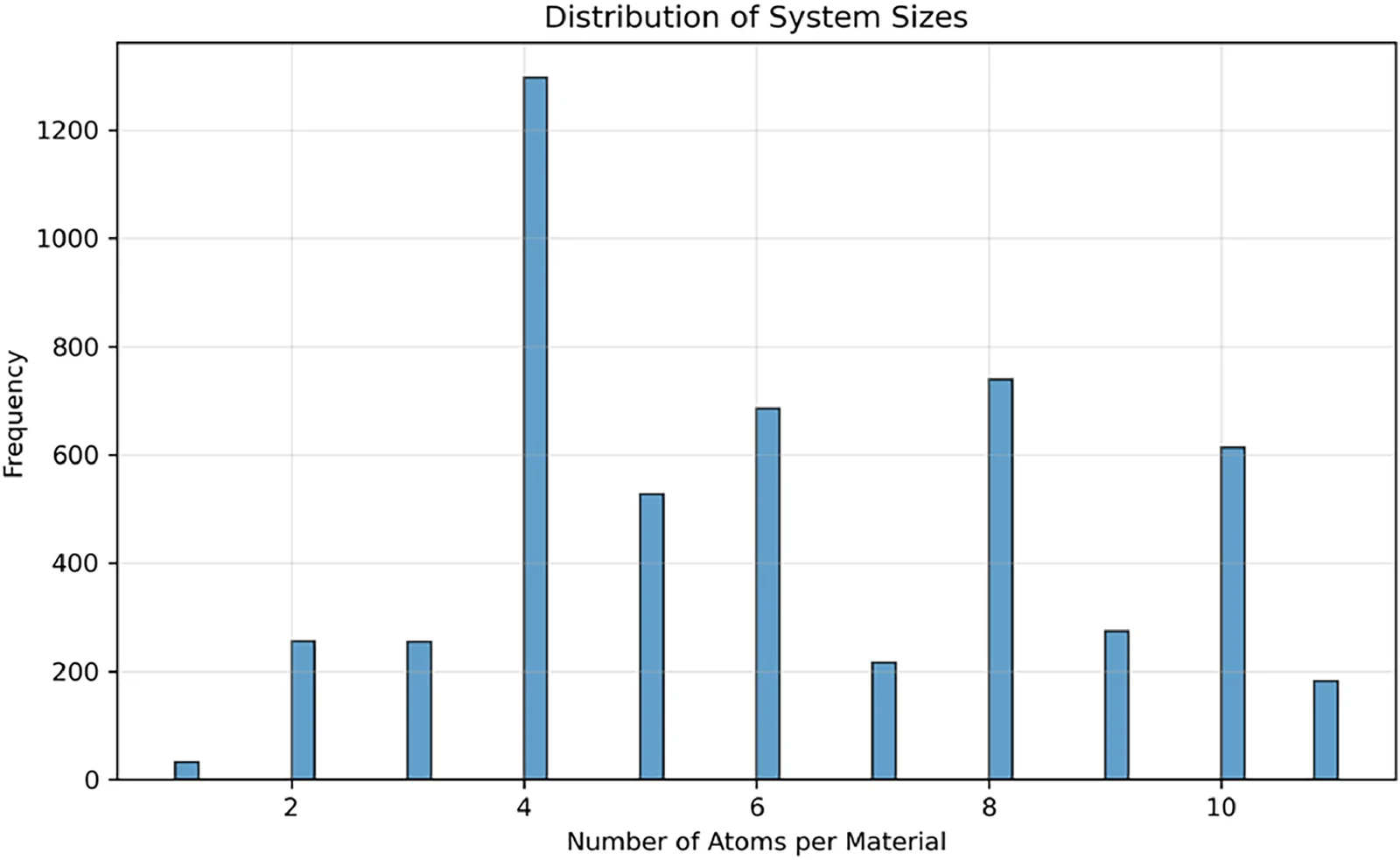
Distribution of atoms per material in the Hirshfeld database. The histogram summarizes the number of atomic sites in each of the 5080 database entries. The entries contain between 1 and 11 atoms, with a median of 6 atoms.

**Fig. 2:**
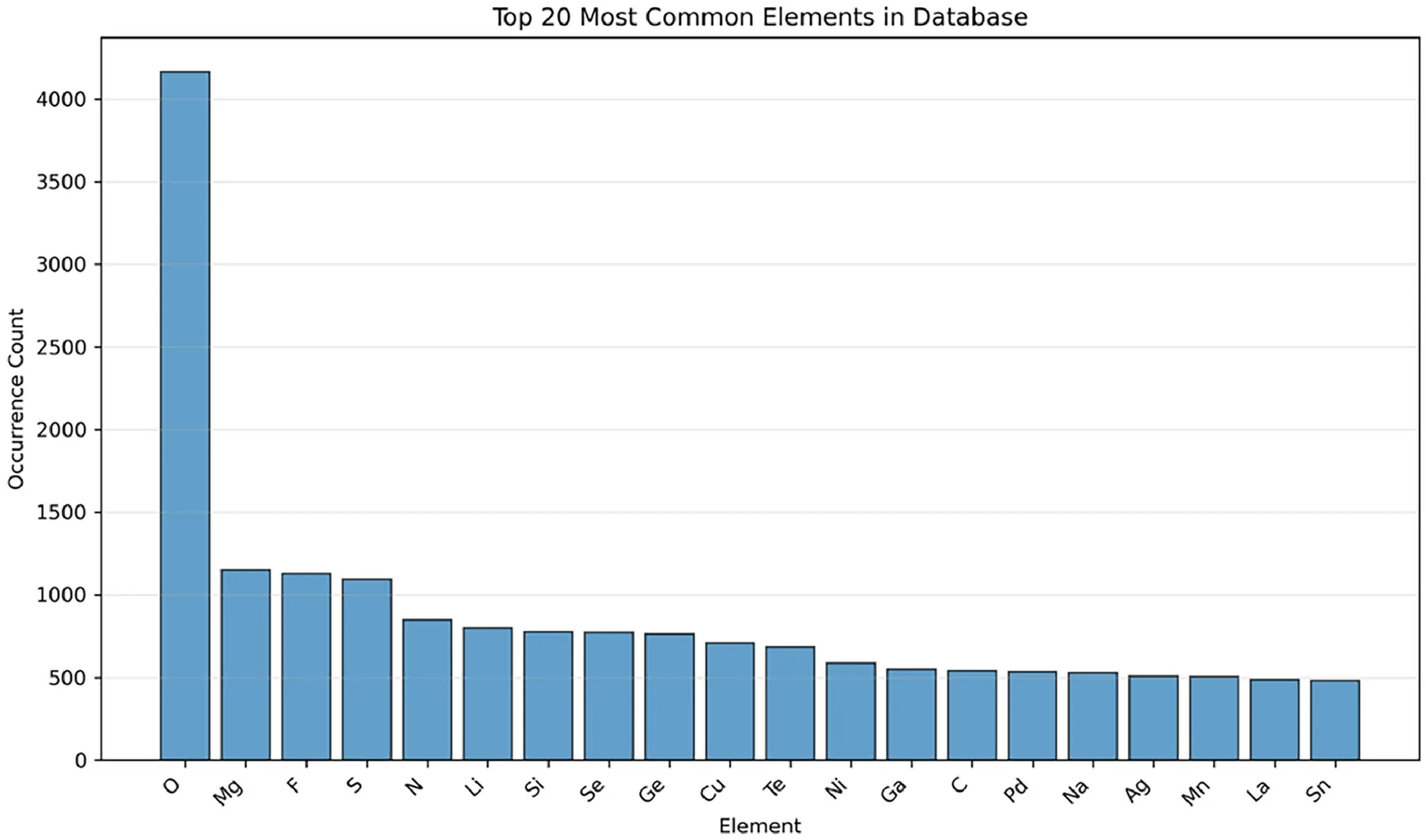
Element abundance in the database. The bar chart shows the 20 most frequent elements among the 31,288 atomic sites. Oxygen is the most common element, followed by Mg, F, S, N, Li, Si, Se, Ge, and Cu.

**Fig. 3:**
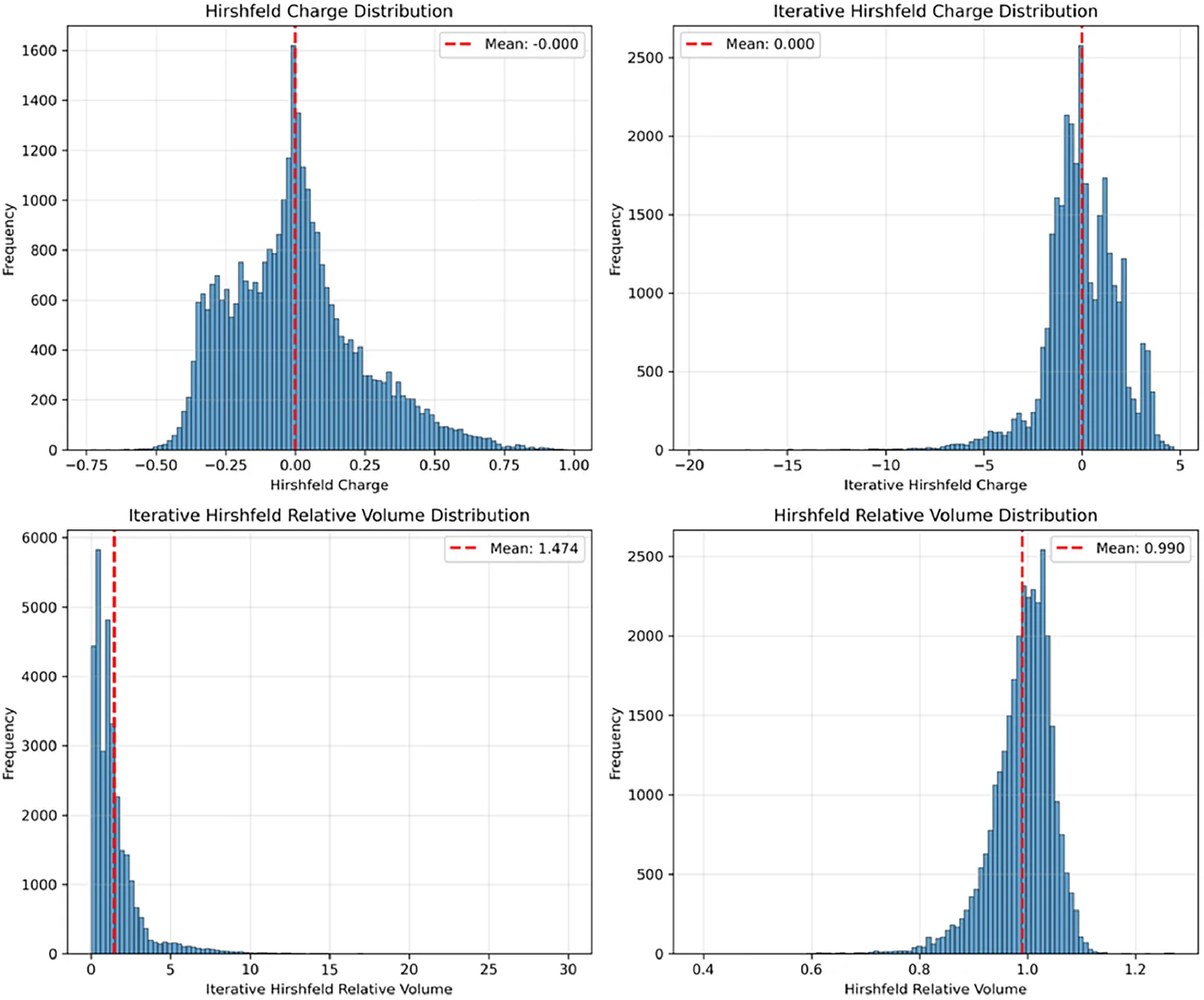
Distributions of the four stored Hirshfeld-related arrays. The panels show atomic-site distributions for standard Hirshfeld charges, iterative Hirshfeld charges, iterative-Hirshfeld relative volumes, and standard Hirshfeld relative volumes.

**Fig. 4:**
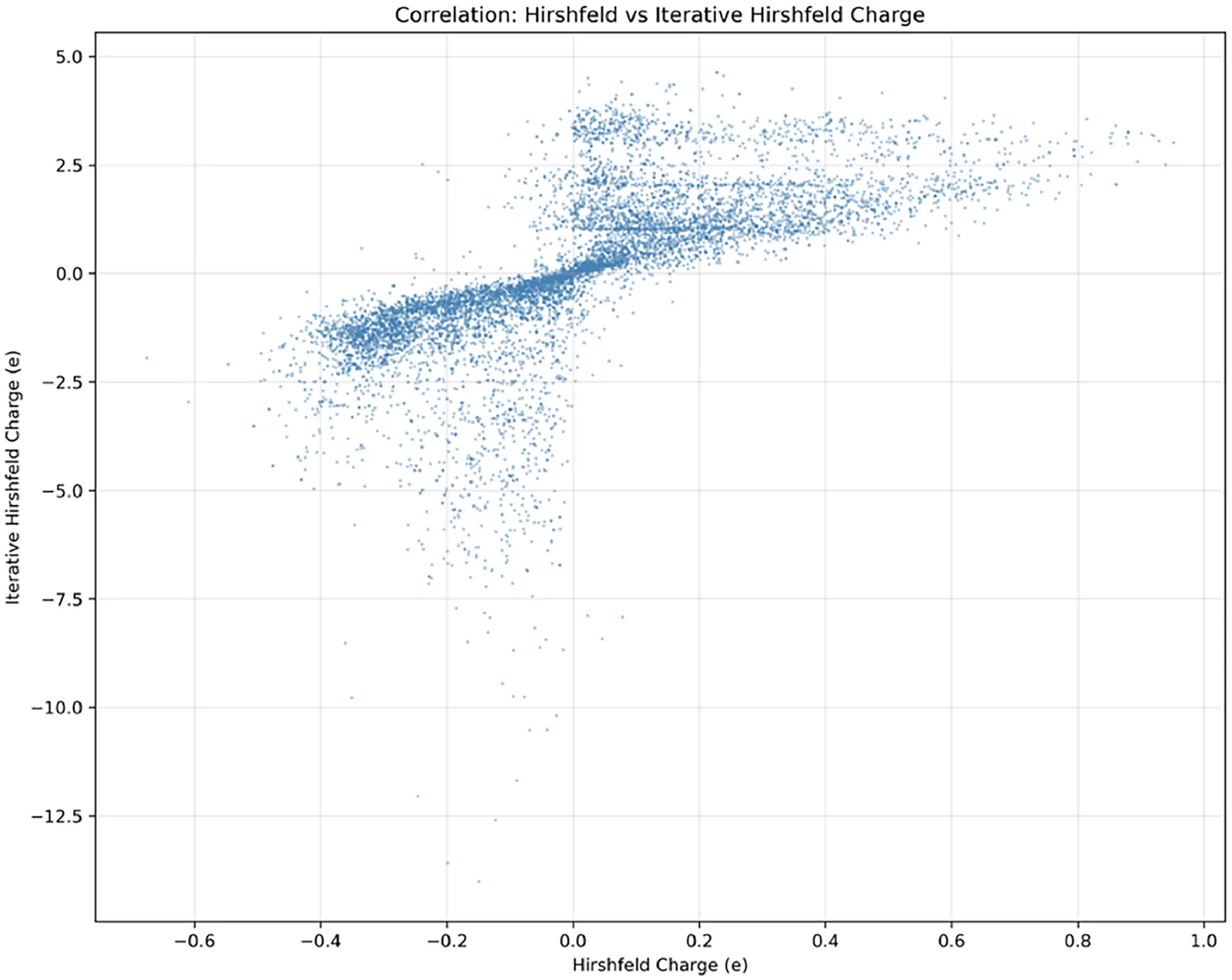
Atomic-site comparison between standard Hirshfeld and iterative Hirshfeld charges. Each point corresponds to one of the 31,288 atomic sites; all sites are plotted, including the extreme iterative-Hirshfeld values. The dashed line marks zero iterative charge. The plot illustrates the moderate relationship and substantially wider range of the iterative partitioning output.

**Fig. 5:**
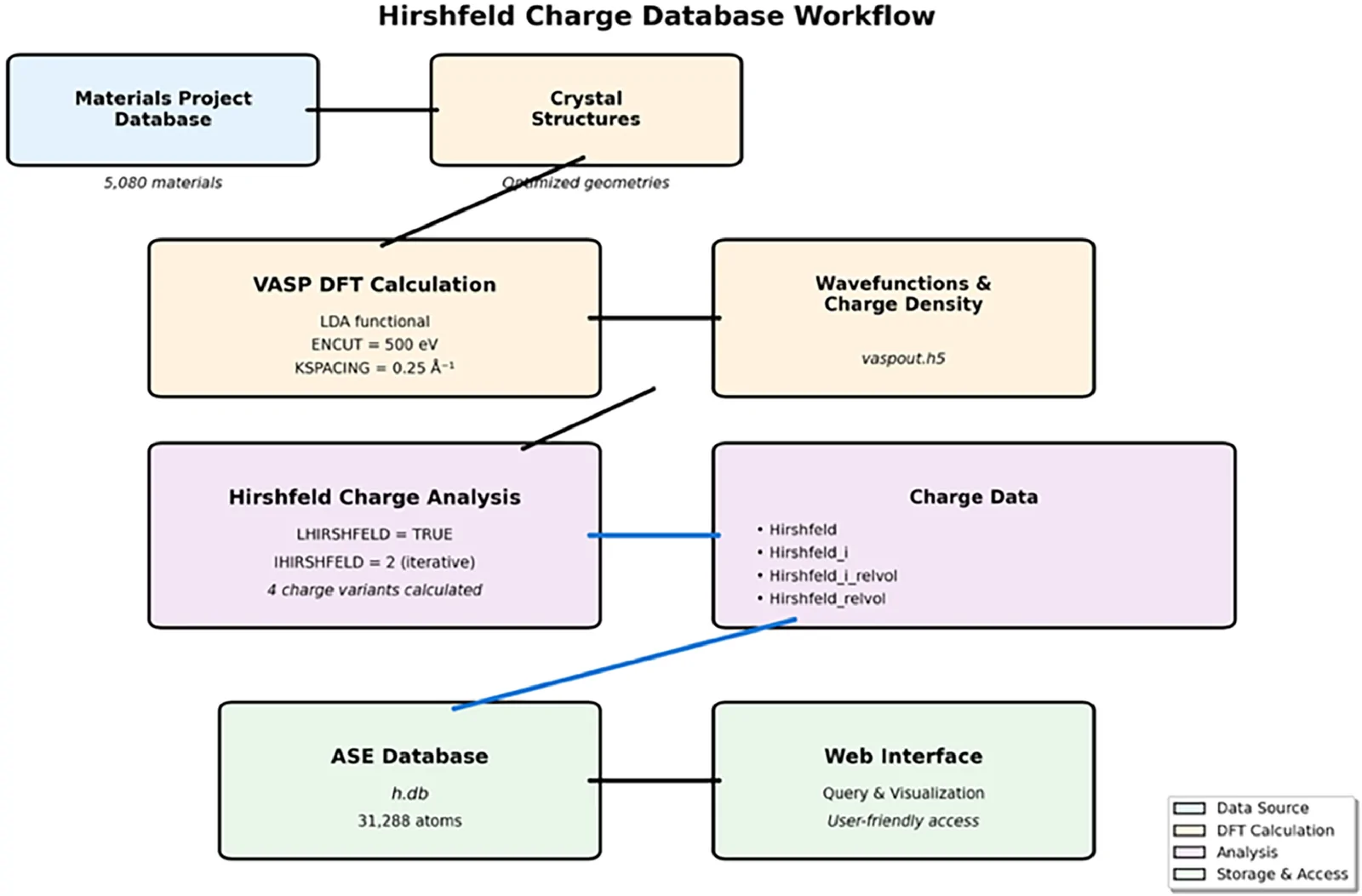
Workflow for generating and packaging the dataset. Materials Project structures are associated with VASP self-consistent-field calculations, Hirshfeld analysis is extracted from HDF5 output, and the resulting arrays are stored in an ASE database with statistics, scripts, figures, and a web-query interface.

**Fig. 6:**
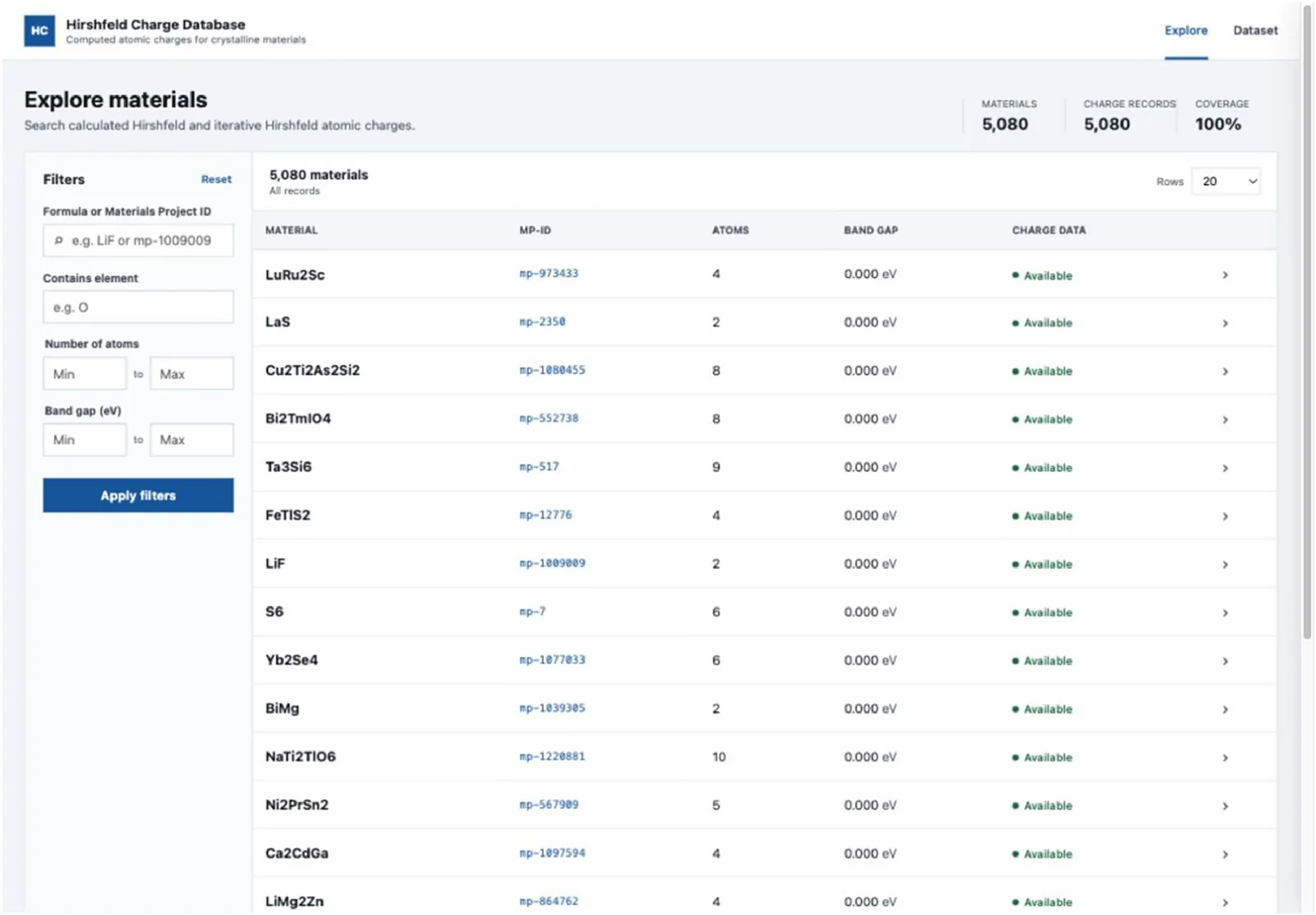
Screenshot of the web-based query interface. The Flask interface included in the data package supports material browsing and search by identifier or formula, enabling users to inspect material-level Hirshfeld descriptors without writing Python code.

**Fig. 7:**
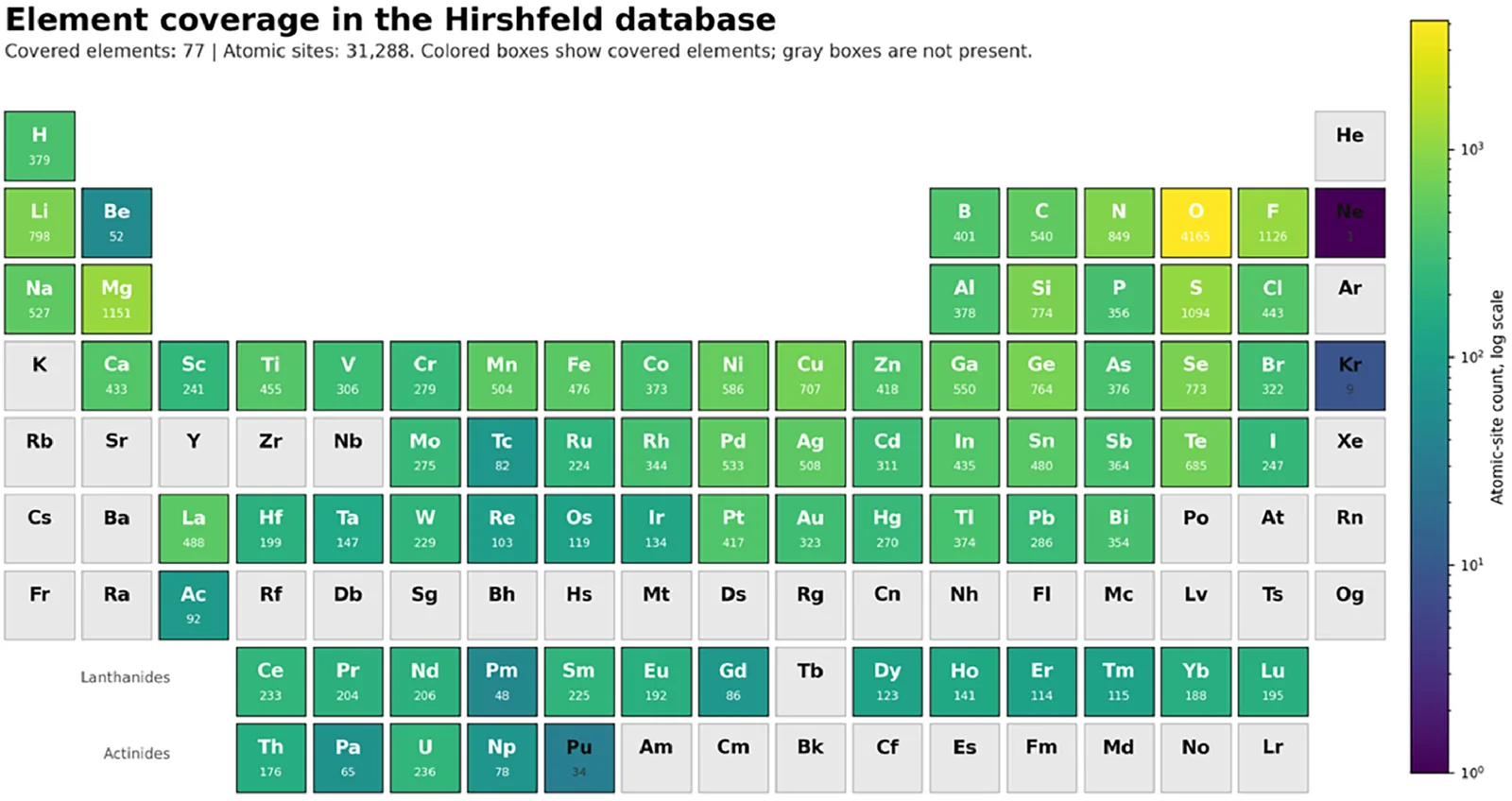
Periodic-table representation of elemental coverage. Colored cells indicate elements present in the 5080-material database, and gray cells indicate elements that are not represented. The number in each colored cell is the atomic-site occurrence count, and the color scale is logarithmic.

Fig. 1 shows the distribution of the number of atoms per material entry. The histogram confirms that the database is concentrated in small unit-cell or primitive-cell entries, with all included entries containing 11 or fewer atoms. Fig. 2 shows the element-abundance distribution for the 20 most common elements. Fig. 3 summarizes the distributions of the four stored Hirshfeld-related arrays. Fig. 4 compares standard Hirshfeld and iterative Hirshfeld charges at the atomic-site level. Fig. 5 gives the data-generation workflow from structure selection to database packaging. Fig. 6 shows the web interface included with the data package. Fig. 7 presents the full elemental coverage on a periodic table, with colors scaled by atomic-site occurrence count.

The accompanying files are organized as follows. The data/ directory contains the main database, summary statistics, database metadata, repository metadata, example table data, element coverage, and calculation parameters. The scripts/ directory contains Python scripts for extracting Hirshfeld data from VASP HDF5 output, checking database completeness, extracting metadata, generating figures, and reproducing the statistical summaries. The figures/ directory contains seven manuscript-ready PNG figures. The compressed file hirshfeld_webgui_20,260,702_190,138.tar.gz contains a Flask web application with the database, templates, static files, start script, and requirements file.

## 4 Experimental Design, Materials and Methods

### 4.1 Materials selection and identifiers

The computational campaign prepared 44,075 Materials Project-indexed candidate directories. The archived release was frozen after the first 5080 successful calculations had been written to the ASE database, using database insertion order as the release order. It is therefore a calculation-availability snapshot rather than a random or stratified sample of the Materials Project, and it is biased toward structures that completed within the campaign and toward small cells (1-11 sites in the archived entries). A material was retained if it had a unique mpid, completed/done/scf workflow metadata, a readable ASE structure, all four required Hirshfeld arrays, and array lengths matching the number of atomic sites. The archived database contains 5080 unique mpid values. Candidate calculations outside this frozen successful-result snapshot, including unfinished or unsuccessful tasks, were not included; their outcome counts were not retained in the archived release metadata.

Release-level validation rejected any candidate row lacking a Materials Project identifier, completed workflow status, one or more required arrays, or matching array lengths. Raw VASP output files are not part of the lightweight repository package; instead, the package provides the extracted Hirshfeld-related arrays, calculation-parameter JSON files, reproducibility scripts, summary statistics, figures, and a web-query interface. Because the database records a unique Materials Project identifier for each entry, users can join these data with additional fields from a versioned Materials Project release.

### 4.2 Density-functional theory calculations

All entries were processed as self-consistent-field calculations using VASP 6.6.0 (6 March 2026 release; build dated 7 April 2026) [12,13] within density-functional theory [14,15]. The local density approximation was used with LDA PAW potentials [16]. The plane-wave cutoff was 500 eV, electronic convergence was 1 × 10^−6^ eV, and Gaussian smearing used ISMEAR = 0 and SIGMA = 0.02 eV. PREC = Accurate, ALGO = All, and NELM = 200 were applied. Reciprocal-space sampling was generated by VASP using KSPACING = 0.25 inverse angstrom. LASPH, LCHARG, LWAVE, and LH5 were enabled. Standard LDA PAW labels were used except for the explicitly archived _sv setups for V, Ca, and Ti.Table 5

### 4.3 Hirshfeld and iterative-Hirshfeld data extraction

For each completed calculation, Hirshfeld outputs were extracted from the VASP HDF5 file at /results/hirshfeldcharge. The extraction script reads four datasets: hirshfeld, hirshfeld_i, hirshfeld_i_relvol, and hirshfeld_relvol. These arrays are converted to Python lists and stored in the ASE database row corresponding to the material mpid. The extraction workflow preserves the original material-level structure and metadata while appending atomic-site Hirshfeld descriptors to the database data field.

The standard Hirshfeld method partitions the electron density using promolecular reference densities. The iterative Hirshfeld procedure updates reference densities until self-consistency in the atomic populations is achieved. The stored relative-volume arrays provide additional partitioning information that can be used to compare atom-in-material volume changes under the standard and iterative procedures.

### 4.4 Database schema and access

*The main database is an ASE database named h.db. It can be read using the ase.db.connect interface:*

**from** ase.db **import** connect

db = connect(``data/h.db'')

row = db.get(mpid="mp-1009,009")

print(row.formula)

print(row.data [``hirshfeld''])

print(row.data [``hirshfeld_i''])

print(row.data [``hirshfeld_relvol''])

print(row.data [``hirshfeld_i_relvol''])

The database key-value pairs include mpid, calc_status, status, jtype, gap, and extra. In the present package all rows have calc_status = done, status = completed, and jtype = scf. The extra field stores JSON-encoded arrays for calculator-derived quantities such as forces, stress, magnetic moments, and dipole when available. The four Hirshfeld-related arrays are stored in row.data.

### 4.5 Quality control and validation

Completeness was checked by traversing all 5080 rows and verifying the four required arrays and their lengths. Workflow-status fields show 5080 entries marked as completed self-consistent-field jobs. These checks verify successful workflow output and database integrity; because per-step electronic residual histories were not retained in the archived database, they are not an independent retrospective audit of every electronic iteration or of Hirshfeld-I convergence behavior. Summary statistics were generated from all 31,288 sites. Charge neutrality was not imposed by the validation scripts. For these charge-neutral calculation cells, the VASP Hirshfeld partition distributes the integrated electron population among the atoms, and the resulting atomic charges sum to zero apart from numerical accumulation error. The maximum residual, approximately 1.8 × 10^−14^ e, confirms material-level charge conservation but does not establish the physical reasonableness of every site value.

### 4.6 Reproducibility files

*The package includes scripts for reproducing the data checks and figures:*

• scripts/extract_hirshfeld.py extracts Hirshfeld arrays from VASP HDF5 output and writes them to the ASE database.
• scripts/check_hirshfeld.py checks whether database rows contain the expected Hirshfeld arrays.
• scripts/extract_metadata.py extracts database schema and representative metadata.
• scripts/detailed_analysis.py inspects database entries and writes example table data.
• scripts/extract_vasp_params.py extracts the VASP settings into JSON.
• scripts/generate_figures.py regenerates the statistical figures.

scripts/analyze_hirshfeld_extremes.py reports non-finite values, iterative-charge threshold counts, and the most extreme site.

The web interface can be unpacked and started with:

tar -xzf hirshfeld_webgui_20,260,702_190,138.tar.gz

cd hirshfeld_webgui_20,260,702_190,138

python app.py

By default, the interface is served at http://localhost:1080. It provides compact database statistics, paginated material browsing, filters for Materials Project identifier, formula, element, atom count, and band gap, and an atom-resolved detail panel for the four stored arrays. The Flask backend exposes JSON endpoints for /api/stats, /api/materials, /api/search, and /api/material/<row_id>. The current interface does not provide a file-download button, CSV export, or a dedicated batch-export endpoint. Users requiring full-database or large-batch extraction should access h.db directly through ASE.

The summed LSF scheduler-reported run time for the 5080 successful calculations was 738,190 s (205.05 h). Assuming that all 40 allocated CPU cores were reserved throughout each job, this corresponds to 29,527,600 allocated core-seconds, or 8202.11 allocated core-hours (341.75 allocated core-days). This is the sum of individual-job run times, not the elapsed duration of the overall campaign; queue waiting time is excluded, and the estimate does not represent measured CPU utilization or energy consumption. Providing the extracted descriptors therefore avoids substantial repeated allocation of computational resources.

## Limitations

The dataset contains quantities generated using one consistent DFT setup. Charge values may differ with other functionals, pseudopotentials, magnetic settings, cell choices, reference populations, or partitioning schemes. The archived set is a successful-calculation snapshot dominated by small cells and is not a statistically representative sample of all Materials Project compounds. Iterative-Hirshfeld values show element- and environment-dependent extremes; they should not be interpreted as formal oxidation states and require outlier review or robust preprocessing before quantitative modeling. For machine-learning use, data should be split at the material level, or by composition/chemical system for stronger extrapolation tests, so atomic sites from one material do not cross training and test sets. Element frequencies are imbalanced, and rare-element predictions require separate validation. The gap key is not a curated electronic band-gap dataset.

## Ethics Statement

This work does not involve human subjects, animal experiments, clinical data, or personally identifiable information.

## CRediT Author Statement

Tianhao Su: Conceptualization, Methodology, Data curation, Formal analysis, Visualization, Writing - original draft. Musen Li: Conceptualization, Software, Data curation, Validation, Visualization, Writing - original draft. Xitao Wang: Validation, Visualization, Writing - review and editing. Shunbo Hu: Methodology, Validation, Writing - review and editing. Tong-Yi Zhang: Supervision, Project administration, Funding acquisition, Writing - review and editing. Tianhao Su and Musen Li contributed equally to this work.
